# An adaptive confidence-driven framework for real-time lidar and visual data fusion in autonomous aerial vehicle landing site assessment

**DOI:** 10.1038/s41598-026-48920-6

**Published:** 2026-05-27

**Authors:** Mudit Raj Sade, Adnan Ahmad, Pranat Saraogi, Muchenedi Hari Kishor

**Affiliations:** School of Computer Science and Engineering, Vellore Institute of Technology, Vellore, Tamil Nadu India

**Keywords:** Autonomous landing site assessment, LiDAR and image fusion, Dynamic environment perception, Safe landing zone detection, Adaptive confidence algorithm, Unmanned aerial vehicles (UAVs), Engineering, Mathematics and computing, Natural hazards

## Abstract

Drones and Aerial Vehicles are now increasingly relying on autonomous landing site assessment to find a safe landing spot. The modern day Technique for finding safe landing site involves use of Both LiDAR and Image data, LiDAR data is used for detecting slope, uneven surfaces, and obstacles like rocks, trees, poles, or buildings based on their 3D structure and image data is used for identifying texture and material understanding. For example – A flat water surface may look safe as per LiDAR data due to its flatness but only through image data we can identify that it is a water surface. Hence it is important to assess safe landing site based on both LiDAR data and image data. The current approach seems to be perfect for static environments but how about in the case of dynamic environments where a safe landing spot identified at a particular time might no longer be safe due to some new obstacle appearance at that spot. This paper addresses this issue by proposing an Adaptive confidence driven algorithm that helps in finding safe landing spots in dynamic or changing environments.

## Introduction

Prior to the adoption of the Hybrid (LiDAR + image-based) methodology for secure landing site identification, algorithms relied solely on either LiDAR or image data for the assessment of landing sites^1,2^. The only LiDAR-based method, as explained in^3^, is a SLAM-based method that uses LiDAR points to figure out the shapes of 3D structures and find safe landing spots and obstacles. When new LiDAR points come in, it updates an existing elevation map, which makes it easier to compute^3–5^. On the other hand, the image-based method mostly uses trained machine learning models to find things in the way^6–8^.

For example, the YOLO model^9^ can be changed to work better with a dataset that has markers showing where it is safe to land and where there are obstacles. This enables the model to identify both in aerial images^10,11^. But this method has some problems.For example, it doesn’t perform well in low light because it can only see things in two dimensions. It cannot quickly figure out the slope, flatness, or height^12–14^.

The hybrid LiDAR + image fusion method solves these problems by combining LiDAR’s 3D structural accuracy with the full visual semantics of image data^18,19^. LiDAR shows the form, slopes, and roughness of surfaces, while photos show texture and material clues to give context^20–22^. This multimodal fusion makes it much easier to find the best safe landing spots, especially in rough terrain^1,23^. This method combines LiDAR point clouds, which show flatness and elevation, with image data that shows detected obstacles and areas that are visually unsafe to make a safety score for each candidate landing patch^5,15^. Zou et al.^15^ employed a SLAM-based methodology similar to^2,3^, where the elevation map is progressively updated rather than entirely reconstructed with each new LiDAR scan. The image processing component employs a CNN-based architecture^6,7,25^ for semantic segmentation and object detection.

The main problem with these current methods is that they assume the environment is always the same^22,23^. In environments that are always changing or dynamic, it’s important to look at safe and unsafe areas that have already been found again^18,19,24^. For instance, imagine a UAV scanning a parking lot: An empty area can be marked as a safe place for a vehicle to land, but once a vehicle arrives and takes up space, it becomes dangerous^1,2^. When a car leaves, an area that was once dangerous may become safe for landing again^21,23^.

To address this problem, we propose an adaptive confidence-driven hybrid framework that dynamically integrates LiDAR and visual (camera) data to assess landing patches in real time^15,17,18^. The LiDAR subsystem makes a 3D point cloud of the ground, fits local planes, and checks slope and roughness to find flat areas with no obstacles^3–5^. The vision subsystem also uses a YOLO-based CNN model to look at pictures and find things that get in the way, like cars, markers, or moving objects^7,9,25^.There have been studies on both deep learning-based methods like YOLO and classical computer vision methods using OpenCV for finding obstacles^26^ when a UAV lands . The incorporation of these multimodal cues ensures improved reliability, adaptability, and situational awareness in decision-making for UAV landings in complex, dynamic environments^1,19,20^.Current LiDAR-vision fusion frameworks for UAV landing predominantly presume static environmental conditions and fail to explicitly account for the temporal validity of landing safety evaluations amid dynamic obstacle introduction. The suggested confidence-driven framework mitigates this deficiency by implementing adaptive temporal reliability modeling for the selection of landing sites.

There has been a lot of research on combining LiDAR and vision in systems for autonomous landing and navigation.Most existing frameworks, on the other hand, presume that once a landing place is declared safe, this classification continues valid until additional observations reveal otherwise. This assumption is true in surroundings that don’t change, but it doesn’t work in situations that do change, where conditions may change between observations.

The major objective of this effort is to modify how we think about landing safety from a permanent label to a transient state. The suggested framework includes a way for confidence to slowly fade away in areas that were previously thought to be safe unless they are re-validated. This means that the safety classification can change. You can look at landing zones again ahead of time with this temporal degradation model, and it stops you from using old environmental data. Section 2 has a structured comparison between the proposed framework and some of the best LiDAR–vision fusion methods to make this difference clearer.Additionally, this work evaluates performance stability through repeated experimental trials, ensuring robustness under dynamic environmental conditions.

## Related work

### LiDAR SLAM for landing site detection

LiDAR based SLAM approach has been used in detecting safe landing spot. In this approach used by^1^, the new LiDAR points are incrementally updated to a global elevation map rather than rebuilding the whole elevation map as new LiDAR data arrives this improves performance by reducing computational overhead. Once the Elevation map is updated, the drone will analyze obstacles, slopes, roughness and then decide on safe landing sites. This approach also makes use of another LiDAR sensor called Navigation Doppler LiDAR (NDL), It measures the Doppler shift in the reflected light to estimate the lander’s velocity relative to the surface. Similar techniques extend to Mars and Moon mission scenarios^16^.

### YOLO for object detection

YOLOv9^9^ is the latest version of the YOLO (You Look Only Once) family which is a real-time object detection Deep Learning model, it processes an input image in a single forward pass through a neural network, predicting bounding boxes and class probabilities. In this latest version two main components have been introduced, firstly PGI (Programmable Gradient Information) which is a mechanism to reduce information loss as the data flows through the deep network. The second feature is Generalized Efficient Layer Aggregation Network (GELAN), it Helps the model remember both fine details and big patterns and ensures that network learns well during the training without forgetting details learnt from earlier layers. This latest version of YOLO is also computationally fast compared to the older versions. This Deep Learning model can be used for obstacle identification during safe landing site detection. In^25^, YOLOv9 was used for safe landing site classification^9^. We can perform such safe landing site detection using the latest version as well. Other work has combined visual detection with depth sensing for landing site hazard avoidance^6^.

### OpenCV-based visual obstacle detection

Using OpenCV to implement classical computer vision methods has been a popular way to find obstacles in real time in robotics and UAV applications. OpenCV-based methods use hand-made visual features like color segmentation, edge detection, contour extraction, and morphological filtering. This is different from deep learning-based models.

Numerous studies have shown that OpenCV’s color-based segmentation can be useful for finding obstacles in controlled settings, especially when there is a clear visual encoding^26^. These methods are computationally efficient and work well for real-time processing pipelines and simulation-based validation where there isn’t much training data.

Even though OpenCV-based methods may not have as much semantic depth as deep learning models, they are good backup options for identifying obstacles and are often used with LiDAR-based geometric analysis in UAV landing systems.

### Hybrid LiDAR–camera fusion for safe landing

The LiDAR + image based (hybrid) technique was used for safe landing site detection as reported in^15^. In this proposed approach the LiDAR data collected from LiDAR scanner and image data was collected from a monocular camera. The LiDAR data collected follows a SLAM based approach for detecting the slope, roughness and obstacle identification for the scanned region. Image data is processed using a Convolutional Neural Network (CNN) for obstacle identification in the image. The image data and LiDAR data are fused together to get more accurate description of the region and to precisely score the landing site and decide if it’s safe to land or not in that region^16^. Multi-modal fusion frameworks for Unmanned Aerial Vehicle (UAV) perception demonstrate the value of combining LiDAR and camera data for improved robustness in changing environments^17^. Recent surveys emphasise multi-sensor fusion for UAV autonomy as essential for dynamic real-world settings^20^.

### Additional LiDAR and vision-based UAV landing approaches

There have been many various implementations of LiDAR and Vision-Based UAV Landing. As reported in^4^ a clear overview of LiDAR technologies for UAV detection with emphasis on navigation and obstacle avoidance was used. As reported in^7^ a hardware-software real-time vision system for automatic UAV landing was developed and it demonstrated how tightly coupled perception and control loops enhance landing precision. As reported in^18^ fusion of visual-inertial odometry and LiDAR-based relative localization to provide cooperative guidance for micro-scale aerial vehicles was implemented . As reported in^1^ emergency landing spot selection algorithms, where multi-modal perception architectures greatly enhance safety under dynamic situations was reviewed. As reported in^21^ the application of UAV photogrammetry along with LiDAR for high-accuracy terrain modeling to assess landing safety in urban settings was implemented.

### Deep learning and semantic understanding approaches

Deep Learning can be used for semantic understanding of the image data for identifying obstacles effectively. As reported in^23^ a deep networks was applied to select landing sites in unstructured environments achieving improved terrain classification. In^22^ semantic understanding for UAV safe landing zones was implemented showing that object-level recognition helps in differentiating safe and unsafe patches. In^19^ multi-modal SLAM combining LiDAR and camera data for robust navigation and landing, emphasizing the value of sensor fusion was implemented. In^5^ LiDAR-guided autonomous landing of aerial vehicles onto ground vehicles demonstrated the benefit of LiDAR in dynamic target acquisition.In^2^ conducted a survey on SLAM-based UAV landing comparing real-world implementations and highlighting practical challenges.

### Advanced depth sensing and texture analysis

Depth sensing techniques have also been combined with monocular-stereo fusion to enhance landing site safety. In^14^ UAV landing in non-cooperative environments using fusion of monocular-stereo depth estimation was demonstrated. In^8^ image segmentation to detect safe landing zones in urban scenarios was implemented . In^10^ used Markov chain-based texture analysis to segment landing areas in aerial images. In^11^ explored UAV landing site detection in urban scenes by identifying roads, vehicles,rooftops,emphasizing context awareness in urban environments. In^12^ introduced advanced method for emergency landing zone detection in UAVs based on a fusion of deep stereo and monocular vision enhanced by attention mechanisms which allows drones to more accurately detect safe landing sites even in challenging or complex environments, improving both the robustness and safety of emergency landings. In^13^ proposed use of monocular vision SLAM (Simultaneous Localization and Mapping) for autonomous UAV landings in emergencies and unknown environments which allows UAVs to perform reliable localization and landing operations without relying on depth sensors like LiDAR, making it suitable for situations where only a single onboard camera is available for further clarity it is tabulated as inTable 1.

**Table 1:** Comparison between existing LiDAR–vision landing frameworks and the proposed confidence-driven approach.

Method	LiDAR–vision fusion	Temporal modeling	Adaptive confidence decay
Zou et al. (2024)^15^	Yes	No	No
Chen et al. (2020)^16^	Yes	Limited	No
Bultmann et al. (2022)^17^	Yes	No	No
Proposed method	Yes	Yes	Yes

## Methodology

### Dataset

This research utilises the TartanAir Neighbourhood Dataset^24^, a comprehensive simulated dataset developed for autonomous navigation, mapping, and landing applications in aerial robotics. It offers high-fidelity RGB, depth, and segmentation data collected in various conditions, rendering it exceptionally suitable for LiDAR–camera fusion research.

#### Dataset overview

Using the AirSim simulation platform, TartanAir is made up of thousands of high-resolution stereo image pairs, depth maps, and ground-truth poses taken from photorealistic virtual environments. The dataset includes a lot of different real-world situations, such as city streets, residential neighborhoods, forests, industrial areas, and open fields. There are different surface geometries, lighting conditions, and moving parts like cars and people in each scene. This variety makes the dataset perfect for testing adaptive confidence-driven landing algorithms in both static and moving environments. The dataset provides synchronized:

- Left and right RGB image sequences (*image_left*, *image_right*),
- Corresponding depth maps (*depth_left*, *depth_right*),
- Ground truth 6-DOF camera poses,
- Optical flow and semantic segmentation maps.

**Fig. 1:**
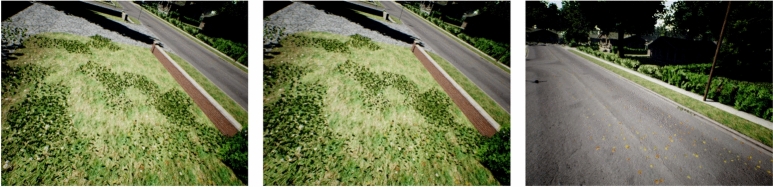
Sample left RGB images from the TartanAir Neighborhood dataset.

Figure 1 shows sample left RGB images from the TartanAir Neighborhood dataset used in this study.In the TartanAir dataset, the term left images refers to RGB frames captured by the left camera of a synchronized stereo camera pair mounted on the UAV. Each left image is temporally aligned with its corresponding depth map, pose, and right image, enabling consistent geometric reconstruction and sensor fusion. In this work, only the left camera stream is used to represent monocular visual perception, while depth information is employed to generate LiDAR-equivalent point clouds for landing site assessment.

### Implementation

This section outlines the comprehensive operational workflow of the proposed confidence-driven landing assessment framework prior to the presentation of the mathematical formulation. Synchronized RGB images and depth maps are employed to reconstruct 3D point clouds via depth-to-point-cloud back-projection. The points that are created are slowly added tomap of the elevation. The confidence values that go with them are added toghether and then lowered over time to show how trust worthy the places that were seen before. After that, the image frame is split into spatial patches, and the geometric scoring from LiDAR and the image-based obstacle scoring are calculated. These ratings are finally combined using weighted integration to find the best landing choice for the current frame. This well-organised pipeline makes sure that spatial and temporal reasoning works in environments that are always changing.

We begin by using image left folder ( left images ) and corresponding left depth maps in depth left folder in the dataset. The depth map of each frame is firstly converted into 3D point cloud data using below formula:

1 $$\begin{aligned} X=\frac{\left( u-c_x\right) \cdot Z\left( u,v\right) }{f_x},\quad Y=\frac{\left( v-c_y\right) \cdot Z\left( u,v\right) }{f_y},\quad Z=Z\left( u,v\right) . \end{aligned}$$

The back-projection from image coordinates to 3D space is performed using Eq. (1).

Here, $(u,v)$ is a pixel’s position.

Each pixel in the depth map encodes the distance $Z(u, v)$ from the camera center to the corresponding point in the scene along the optical axis. To reconstruct the 3D structure of the scene (i.e., obtain a *point cloud*), the intrinsic calibration parameters of the camera are used. These parameters describe how 3D world points are projected onto the 2D image plane.

The pinhole camera model defines the relationship between a 3D point $(X, Y, Z)$ in the camera coordinate system and its image projection $(u, v)$ as:

2 $$\begin{aligned} u = f_x \frac{X}{Z} + c_x, \qquad v = f_y \frac{Y}{Z} + c_y \end{aligned}$$

The forward projection from 3D camera coordinates to the image plane is defined by Eq. (2).

where $f_x$ and $f_y$ are the focal lengths (in pixel units) along the horizontal and vertical axes, and $(c_x, c_y)$ represents the principal point, i.e., the location where the optical axis intersects the image plane.

By rearranging the above equations, we obtain the equations in (1) that recover the 3D coordinates from pixel coordinates and the corresponding depth value $Z(u,v)$:

Each pixel $(u, v)$ in the depth map can therefore be back-projected into the 3D space of the camera. The resulting set of all such points forms a dense 3D point cloud representation of the observed scene, where:

- $X$ denotes the horizontal position of the point (in meters) relative to the camera center,
- $Y$ denotes the vertical position, and
- $Z$ represents the distance (depth) from the camera.

Next, this new 3D point is mapped to corresponding cell in the elevation map. If this cell was previously not scanned, then it adds this new height to LiDAR elevation map and assigns an initial confidence weight in the confidence map which is a 2D grid that shows how certain or reliable the elevation data is for each region, using accumulated LiDAR scans. Each cell’s confidence increases when new LiDAR points are added and decreases or decays if the region isn’t scanned for a while. The updates done to the elevation map is similar approach that happens in LiDAR SLAM^3^. But, if the cell has already been scanned before then the height is updated in the LiDAR elevation map using below formula:

3 $$\begin{aligned} h_{\text {new}}=\frac{w_{\text {old}}\cdot h_{\text {old}}+w_p\cdot Z_i}{w_{\text {old}}+w_p} \end{aligned}$$

The elevation value update for a previously scanned cell is computed using Eq. (3).

Here,

- $h_{\text {old}}$ is the previously stored height value in the elevation map,
- $w_{\text {old}}$ is the accumulated confidence (or weight) of that cell based on past LiDAR observations,
- $Z_i$ is the height of the newly projected 3D point from the current depth frame, and
- $w_p$ is the confidence weight assigned to this new point.

And the weight (confidence) is updated using below formula:

4 $$\begin{aligned} w_{\text {new}}=w_{\text {old}}+w_p \end{aligned}$$

The confidence accumulation for each elevation cell follows Eq. (4).

$w_p$ is typically set to 1, and $w$ is decayed over time to keep the model adaptive:

5 $$\begin{aligned} w \leftarrow \alpha \,w \end{aligned}$$

Temporal decay of confidence values is applied using Eq. (5).

Along with the discrete multiplicative decay formulation in Eq. (5), the temporal confidence evolution can also be thought of as an exponential decay process in continuous time:

6 $$\begin{aligned} C(t) = C_0 e^{-\lambda (t - t_0)} \end{aligned}$$

$C_0$ is the confidence value at reference time $t_0$, *t* is the current time, and $\lambda$ is the environmental volatility factor. The parameter $\lambda$ determines how quickly previously seen landing areas lose reliability if they aren’t looked at again. When $\lambda$ is high, it means that the environment is very dynamic; when it is low, it means that the environment is relatively static.

The UAV’s speed also affects the effective decay rate from a motion point of view. The faster the UAV moves, the faster regions leave the sensor’s field of view, which makes it harder to know how safe they are right now. So, the decay factor $\lambda$ can be thought of as being related to the UAV’s speed *v*:

7 $$\begin{aligned} \lambda = k v \end{aligned}$$

where *k* is a number that doesn’t change based on the sensor range or how the environment changes. This equation makes a direct mathematical connection between how fast a car goes and how sure it is of itself.

This work’s confidence formulation does not signify probabilistic occupancy or mapping uncertainty in the traditional SLAM context. In contrast to occupancy grid models, which utilize Bayesian updates and normalization constraints to estimate the probability of obstacle presence, the proposed confidence values do not constitute probability distributions and are not obligated to total one. The confidence term shows how reliable the most recent observed state of a landing area is over time. Confidence accumulation (Eq. 4) denotes the aggregation of consistent observations, whereas multiplicative decay (Eq. 5) facilitates the gradual obsolescence of outdated information when areas are not looked again. This formulation is deliberately crafted as a temporal validity mechanism for dynamic landing evaluation rather than a probabilistic mapping framework.

Next, the corresponding image frame is divided into certain number of patches depending on the Patch size and the LiDAR score and image score is calculated for each of these patches.

#### LiDAR scoring for a patch

For LiDAR-based scoring, we project the corresponding new LiDAR points onto the image to consider only those LiDAR points that lie inside that patch. Each LiDAR point is described by 3D coordinates $(X, Y, Z)$ and is projected onto the 2D image using the camera intrinsic parameters $f_x,\;f_y,\;c_x,\;c_y$. The projection is given by:

8 $$\begin{aligned} u=\frac{f_x\,X}{Z}+c_x, \quad v=\frac{f_y\,Y}{Z}+c_y. \end{aligned}$$

The projection of 3D LiDAR points onto the image plane is computed using Eq. (6). This projection presupposes that the LiDAR and camera coordinate frames are extrinsically calibrated and temporally synchronized. The transformation between LiDAR and camera frames in the simulation environment is predetermined and established, guaranteeing uniform spatial alignment during patch-wise evaluation. In above $(u,v)$ represent the location in the image where the 3D point would appear. To check if a LiDAR point falls inside an image patch the projected image coordinates are compared against the patch bounds. Only those LiDAR points whose projections fall within the patch are used for the scoring of that patch.

After that we fit a plane to the LiDAR points in the patch using SVD (Singular Value Decomposition). SVD is employed to determine the plane that optimises the squared perpendicular distances of all points from that plane (a least-squares fit). This is a strong and numerically robust method to estimate the parameters of the plane (normal vector and centroid) which are later used to determine the slope and roughness of the terrain^16^.

Centroid calculation:

9 $$\begin{aligned} \textbf{c}=\frac{1}{N}\sum _{i=1}^{N}\textbf{p}_i \end{aligned}$$

The centroid of LiDAR points within a patch is calculated using Eq. (7).

$N$ is the number of LiDAR points in the patch and $\textbf{p}_i$ represents the 3D coordinates of the LiDAR point.

Centering points around their mean translates their coordinate system to the centroid of the point-cloud. This removes the translational contribution and highlights the distribution over space. It accounts for point spread with respect to the centroid. Centering enables SVD to compute principal directions such that the smallest singular vector is the plane normal.

10 $$\begin{aligned} q_i = p_i - c \end{aligned}$$

Centering of LiDAR points relative to the centroid is performed using Eq. (8).

$(q_i)$ is a column vector.

Now creating a column matrix of all these $(q_i)$ points:

11 $$\begin{aligned} M = \begin{bmatrix} q_1^T \\ q_2^T \\ q_3^T \\ \vdots \\ q_N^T \end{bmatrix} \end{aligned}$$

Single value decomposition of matrix $M$:

12 $$\begin{aligned} M = U \Sigma V^\top \end{aligned}$$

The singular value decomposition of the centered point matrix is expressed in Eq. (10).

$U$ is an $(m\times m)$ orthogonal matrix whose columns are called the left singular vector of $M$. $\Sigma$ is a diagonal matrix containing the singular values of $M$, which are non-negative real numbers arranged in descending order along the diagonal. It represents the scaling factors. $V$ is an $(n\times n)$ orthogonal matrix whose columns are the right singular vector of $M$.

13 $$\begin{aligned} \textbf{n} = V_{[:,-1]} \end{aligned}$$

The surface normal vector is obtained from the SVD result as shown in Eq. (11).

$\textbf{n}$ is the normal vector which is the last column of $V$.

14 $$\begin{aligned} d_i = \left( p_i - c\right) \cdot \textbf{n} \end{aligned}$$

The perpendicular distance of each LiDAR point from the fitted plane is computed using Eq. (12).

Distance of a LiDAR point $p_i$ from the plane that is fitted to the LiDAR points in the patch using SVD.

RMS (Root Mean Square Distance) from the plane:

15 $$\begin{aligned} \textrm{rms} = \sqrt{\frac{1}{N} \sum _{i=1}^N d_i^2} \end{aligned}$$

Surface roughness within a patch is quantified using the RMS metric defined in Eq. (13).

RMS calculates the average deviation of the 3D points from their estimated best-fit plane. The perpendicular distance $d_i$ for each point $i$ shows how far that point is from the fitted plane surface. The RMS value adds up these distances for all $N$ points in the patch. A lower RMS value means that most of the points are close to the plane, which means that the surface is mostly flat and smooth. On the other hand, a higher RMS value means that the points are at very different heights, which means that the area is rough, uneven, or cluttered and may not be good for a stable landing.

Slope angle calculation:

16 $$\begin{aligned} \theta = \arccos \left( \textbf{n} \cdot \textbf{k}\right) \end{aligned}$$

The slope angle between the surface normal and the vertical axis is computed using Eq. (14).

Here $\textbf{n}$ is the surface normal vector, $\textbf{k}$ is the vertical unit vector.

17 $$\begin{aligned} {\textrm{slope}}_{\textrm{deg}} = \frac{180}{\pi } \times \theta \end{aligned}$$

The slope angle in degrees is obtained using Eq. (15).

The geometric score is calculated as:

18 $$\begin{aligned} \textrm{score} = {\textrm{slope}}_{\textrm{deg}} + 100 \times \textrm{rms} - \frac{N}{10000} \end{aligned}$$

The geometric suitability score for a landing patch is computed using Eq. (16).

The above geometric score is multiplied by the mean of confidence values of the points in that patch in the confidence map, the reason being confidence values indicate how recently this patch has been evaluated, if a patch has been evaluated multiple times then it means there is certain surety about this patch’s current state and with time these confidence values of every cell in the confidence map also decay thus reducing surety/certainty of the current state of that particular patch/location that those cells in the confidence map make up.

19 $$\begin{aligned} \mathrm {LiDAR\_score} = \max \bigl (0,\;100-\textrm{score}\bigr )\times \mathrm {mean\;Confidence} \end{aligned}$$

The final LiDAR-based safety score is calculated using Eq. (17).

#### Image scoring in a patch

We chose YOLOv9 for detecting obstacles because it works better and faster than other real-time UAV perception tasks. It has Programmable Gradient Information (PGI) and the Generalised Efficient Layer Aggregation Network (GELAN), which work together to keep track of both the big picture and the small details. This makes it possible to accurately find small or partially hidden obstacles like rocks, cars, or poles, even in scenes that are busy and changing^9^.Also, its ability to make real-time inferences makes sure that obstacle detection stays fast during in-flight landing evaluations. This is important for making decisions that change based on the situation.

While newer real-time detectors such as YOLOv10 provide improved efficiency on edge devices, YOLOv9 was selected in this work due to the availability of stable pretrained models and its widespread adoption at the time of implementation. Importantly, the proposed framework is detector-agnostic and does not rely on any specific neural network architecture for obstacle identification. This is further demonstrated in the AirSim-based simulation, where OpenCV-based visual processing is employed instead of deep learning models, thereby reducing detector dependence and highlighting the generality of the confidence-driven fusion mechanism.

In a particular patch, once YOLO has identified obstacles then all those LiDAR points that do not lie on any obstacles are considered for Image scoring.

20 $$\begin{aligned} \mathrm {image\_score} = \frac{\mathrm {number\;of\;obstacle-free\;LiDAR\;points\;in\;patch}}{\mathrm {total\;number\;of\;LiDAR\;points\;in\;patch}} \times 100 \end{aligned}$$

The image-based safety score for each patch is defined in Eq. (18).

21 $$\begin{aligned} \mathrm {final\_score} = \mathrm {LiDAR\_WEIGHT} \times \mathrm {LiDAR\_score} + \mathrm {IMAGE\_WEIGHT} \times \mathrm {image\_score} \end{aligned}$$

The final fused landing suitability score is computed using Eq. (19).

Now for all patches this final score is calculated and the one with the highest score is considered to be safe landing site in that image frame and is hence marked.

But the part that this paper is trying to propose has got more to deal with LiDAR part than with the image part. This paper is proposing an approach that can be useful in dynamic environments. The confidence values in the confidence map increase indicating that certainty of the state of that region has increased whenever a particular region is revisited or has appeared in a scan and decrease/decay if that region has not been scanned recently again increasing the uncertainty of the state of that region as time passed by. It is also important to note that confidence values in confidence map do not say anything about safety of a region, they just indicate the certainty of the state of a region at a point of time. The way this approach works can be explained with an example like – Let’s imagine a scenario in which a drone is trying to land in a place where different obstacles are present like trees, vehicles, water well etc., it started scanning from a particular point at a particular time it found a spot that is safe to land but it will continue scanning up to some time because it may find an even better space that is further ahead as time passes some obstacle say truck appears in that spot and at that time the drone is still scanning for other safe spot and that previous region is out of the LiDAR range and it found some safe space to land but it’s not as safe as the previous one so it will decide to go to land in that previous spot, that spot is out of LiDAR range it does not know about its current state any more, so in this case if the drone made use of the adaptive confidence approach, the confidence values of the previously identified safe spot would decay over time and the drone might not remember that there was a safe spot identified previously so it will have to scan for current state of that region again to get the exact current state of that spot and reassess if it’s safe to land there or not. This can be useful in scenarios where there is a momentary LiDAR scanner failure due to which drone is no longer able to scan the landing spot any more or when scanned region is out of LiDAR scan bounds to be scanned again for state of that region, where reliance on already collected data is not safe as the state of the landing spots detected may have changed^16,17^. YOLOv9 has a number of architectural improvements over older object detection models like YOLOv5 and YOLOv8 that make both detection accuracy and train- ing stability much better. Programmable Gradient Information (PGI) is one of the most important new features. It lets you regulate how gradient information moves through the network. In deep neural networks, backpropagation can cause information to degrade, which can make it harder to recognise small objects. PGI helps with this problem by keeping important gradient components, which lets the model keep both low-level spatial details and high-level semantic properties while it trains. The Generalised Efficient Layer Aggregation Network (GELAN) is another impor- tant part of YOLOv9. It makes it easier to combine features from multiple layers. GELAN is better than the old feature pyramid networks used in prior versions of YOLO because it allows for more efficient multi-scale feature fusion without adding a lot of extra computation time. This leads to better representations of items at differ- ent scales. This is especially relevant in aerial images, where things like cars, poles, or debris may look small, partially hidden, or at different distances from the UAV. On the other hand, YOLOv5 uses typical CSP-based designs and PANet to combine features. This is efficient, but it may not keep gradients as well in deeper layers. With better architecture and anchor-free detection, YOLOv8 is better than this, but it doesn’t directly address gradient information flow as well as PGI does in YOLOv9. Because of this, YOLOv9 is more stable in complicated environments with clutter, occlusion, and changing lighting conditions. Also, YOLOv9 can still make inferences in real time even with these improvements, which makes it perfect for applications that need to happen quickly, like landing an autonomous UAV. In these situations, quick and accurate obstacle identification is necessary for making safe choices. 

### Theoretical framework

The suggested confidence-driven fusion framework can be understood through a probabilistic decision-theoretic lens. In classical occupancy grid mapping, each cell shows the likelihood of an obstacle being present. In this work, the confidence value shows how reliable the landing safety validity is over time.

Let *S* represent the occurrence of a safe landing patch at time *t*. The fused safety decision relies on a confidence-weighted score that estimates:

22 $$\begin{aligned} P(S|Z_t, I_t, \mathcal {H}_{t-1}) \end{aligned}$$

Here, $Z_t$ signifies LiDAR observations, $I_t$ signifies image observations, and $\mathcal {H}_{t-1}$ denotes historical observations represented by aggregated confidence values.

Confidence decay adds a time-based prior that limits how much you can trust past observations. This makes sure that the effects of past measurements fade away quickly over time if they aren’t confirmed again. This stops old safety assessments from affecting current decisions.

In contrast to classical SLAM-based probabilistic mapping methods, the fusion mechanism gives bounded temporal trust instead of just estimating spatial occupancy.

#### Distinction from Probabilistic Occupancy Grid Models

The proposed confidence formulation does not signify probabilistic occupancy or mapping uncertainty as typically employed in SLAM-based occupancy grids. This study quantifies the temporal reliability of previously evaluated landing safety through confidence values. In contrast to occupancy probabilities that assess the likelihood of obstacle presence, the proposed confidence metric quantifies the temporal validity of landing suitability. The decay mechanism functions as a temporal invalidation strategy specifically designed for dynamic landing evaluation rather than probabilistic spatial mapping.

## Experimental setup

### Simulation environment

We tested the suggested solution in a high-fidelity simulation environment that used AirSim and Unreal Engine together. AirSim lets you realistically simulate flying planes, onboard sensors, and environments that change all the time. This makes it useful for testing of autonomous landing scenarios in a controlled way.

The UAV was initialized using the AirSim Multirotor API and operated under controlled flight conditions. It followed a predefined raster scan trajectory over the environment at a fixed altitude of approximately 8 meters, ensuring uniform spatial coverage of the region. The simulated UAV platform had a 3D LiDAR sensor and an RGB camera, which are common tools for evaluating landing sites for autonomous vehicles. The UAV followed a set raster-scan path at a set height during each experiment. This made sure that the environment was covered in an orderly way and that the sensor perspectives were the same across frames.

The simulation took place in a 10 × 10 m flat area (ranging from −5 m to +5 m in both X and Y directions), discretized using a grid resolution of 0.25 meters. The environment was populated with cone-shaped obstructions representing hazardous landing zones. Each cone had an approximate height of 2.0 meters and a base scaling of 0.6 × 0.6 meters, simulating real-world obstacles such as poles or small structures. Three candidate landing zones were defined as circular regions with a radius of approximately 0.7 meters. Initially, all landing zones were obstacle-free. To test temporal safety reasoning, dynamic obstacles were deliberately introduced at predefined time steps: an obstacle was inserted in the first landing zone at frame 60, and another in the third landing zone at frame 110, while the second region remained obstaclefree throughout the experiment. Three experimental setups were carried out under this uniform environmental and dynamic conditions to guarantee an unbiased comparative assessment. Mode 0 (LiDAR-only baseline) only used geometric scoring and didn’t have any visual input or temporal confidence modelling. Mode 2 (Fusion without confidence decay) combined LiDAR and vision and turned off temporal confidence decay. Mode 1, which is the method we suggest, used full LiDAR-vision integration along with adaptive confidence accumulation and decay. To make sure the comparison was fair and could be done again, all modes used the same UAV path, obstacle insertion schedule, and decision horizon. We ran each experiment six times (n = 6) to make sure the results were statistically strong and to take into account the fact that people see things differently. To make sensor observations and decision scoring more like noise in the real world, we added small random changes to them. This was done to make it look like sensors were uncertain and the environment was changing. All of the runs used the same courses and times for the UAVs to add obstacles. This made it fair and controlled to compare the modes. The mean and standard deviation were used to combine the results so that they would give a statistically accurate picture of how well the system worked.

### Sensor configuration

The simulated UAV had a 3D LiDAR sensor that took dense point cloud measurements of the ground and nearby obstructions, and an RGB camera that took pictures of the surroundings. LiDAR readings were utilised to analyse the shape of the surface and the presence of obstacles, while RGB photos were used to visually check that the landing was safe.

Both sensors had set update rates and were synchronised in time, so that each LiDAR point cloud matched the same scene state as the RGB image that went with it. It was expected that the extrinsic calibration between the sensors was fixed and known. This made sure that the LiDAR grid cells and picture patches were always in the same place during processing.

### Software and processing stack

The simulation and processing pipeline was built using a modular software design. AirSim took care of making models of the environment, simulating physics, and creating sensor data. The proposed confidence-based landing assessment method was built using a processing framework based on Python.

We divided LiDAR point clouds into spatial grid cells and kept a confidence value for each cell. Repeated, consistent observations made people more sure of themselves, but over time, without confirmation, they became less sure of themselves, which made once-safe areas less safe.

OpenCV was used to process RGB image frames in the AirSim-based simulation to find and check for obstacles and make sure it was safe to land. Image-based analysis made it possible to find areas that were visually risky. This added a semantic safety score to the overall confidence-driven landing assessment framework. We used NumPy for maths and Matplotlib to show confidence maps and heatmaps across simulation frames while we weren’t connected to the internet.

All tests were done on a computer with an intel core i9, an RTX 5030 GPU, and 16 GB of RAM.

The implementation was made with Python (version 3.10), OpenCV, NumPy, and Windows AirSim. These tools make assurance that the experimental procedure can be repeated.

### Obstacle modeling

To show risky landing regions, cone-shaped geometric primitives were scattered out all over the terrain. These primitives show physical barriers that make landing areas risky. We added dynamic impediments by adding more cones in some possible landing zones after the UAV had previously scanned those sites. This technique is like what happens in real life when areas that look safe at first might become risky later because of vehicles, equipment, or other temporary obstructions.

**Fig. 2:**
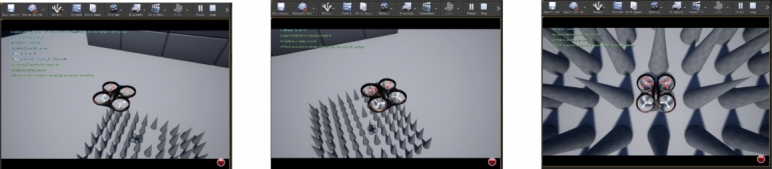
AirSim-based simulation environment illustrating the UAV platform and dynamically introduced cone-shaped obstacles representing unsafe landing regions.

The controlled timing and placement of dynamic obstacles allowed for an accurate assessment of the suggested confidence decay mechanism and its capacity to dismiss landing sites whose safety validity diminishes over time. This experimental setup was maintained consistently across the 6 independent runs, ensuring that observed performance variations arise from stochastic perception effects rather than changes in environmental conditions as shown in the figure 2.

**Fig. 3:**
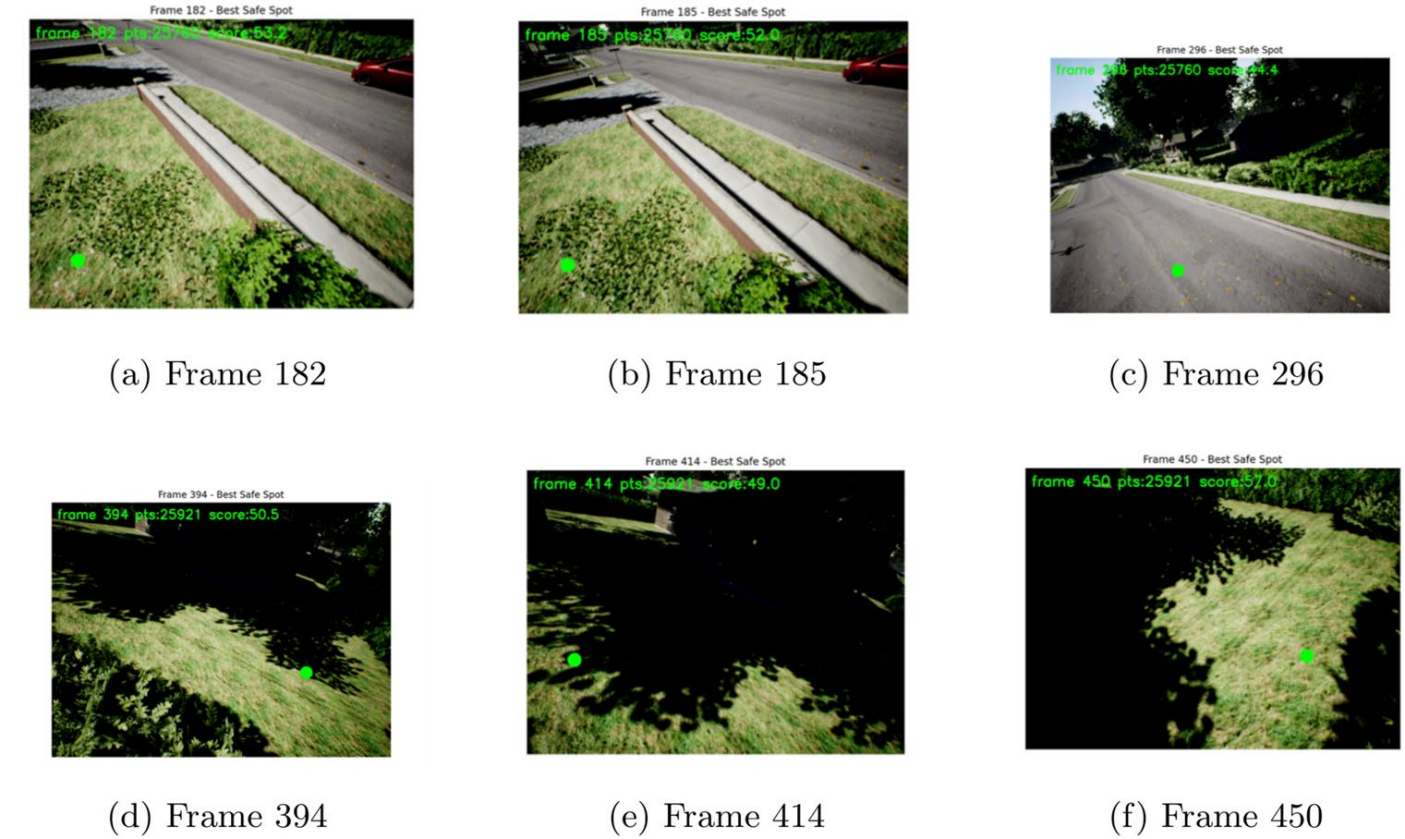
safe spots in few of the image frames from frame no. 182 till frame no. 450.

Figure 3 shows representative views of the AirSim-based simulation environment used to evaluate the proposed confidence-driven landing assessment framework.

## Results

### Dataset-based evaluation using TartanAir

We have given 268 frames of LiDAR data and its corresponding image data as input and got all the safe landing spots marked in each of those frames. We also obtained the Global Elevation Heat map and Confidence Heat map. For implementation we used the following variable values:

- LiDAR WEIGHT = 0.7
- IMAGE WEIGHT = 0.3
- PATCH SIZE = 160

Confidence values decay after processing every 10th frame. We got safe spots in every image frame from frame no. 182 till frame no. 450. A few of them are shown below:

Figure 3 presents representative image frames illustrating the detected safe landing spots across different time instances. Figure 3 illustrates the consistency of detected safe landing regions across multiple frames despite changes in viewpoint and sensor perspective. The selected landing patches remain spatially stable over time, indicating robustness of the LiDAR–vision fusion and confidence-weighted scoring mechanism prior to the introduction of dynamic obstacles. This qualitative consistency motivates the subsequent analysis of temporal confidence evolution under changing environmental conditions.

We also got the Elevation map and confidence Map. The Elevation map represents how when new LiDAR points are coming and how they are being updated or added to the map. The confidence map represents the confidence values of every cell in the elevation map. It is important to note that the Elevation map and the Confidence map involve the information of all the frames processed till then rather than one frame.

Figures 4, 5, 6, 7, 8, 9, 10, 11, and 12 illustrate the evolution of the elevation and confidence maps after processing different numbers of frames.

**Fig. 4:**
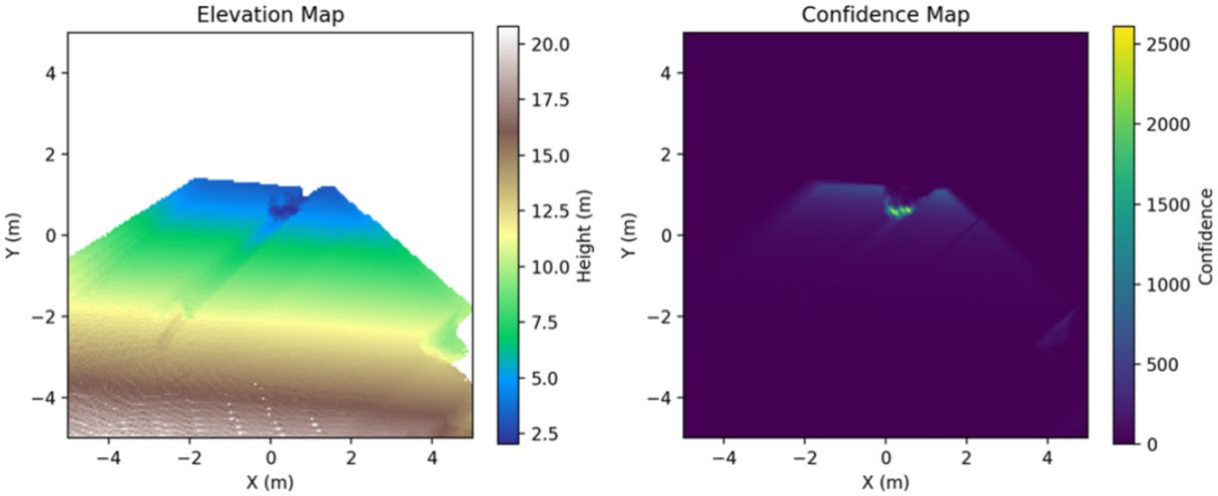
After processing till Frame 185.

**Fig. 5:**
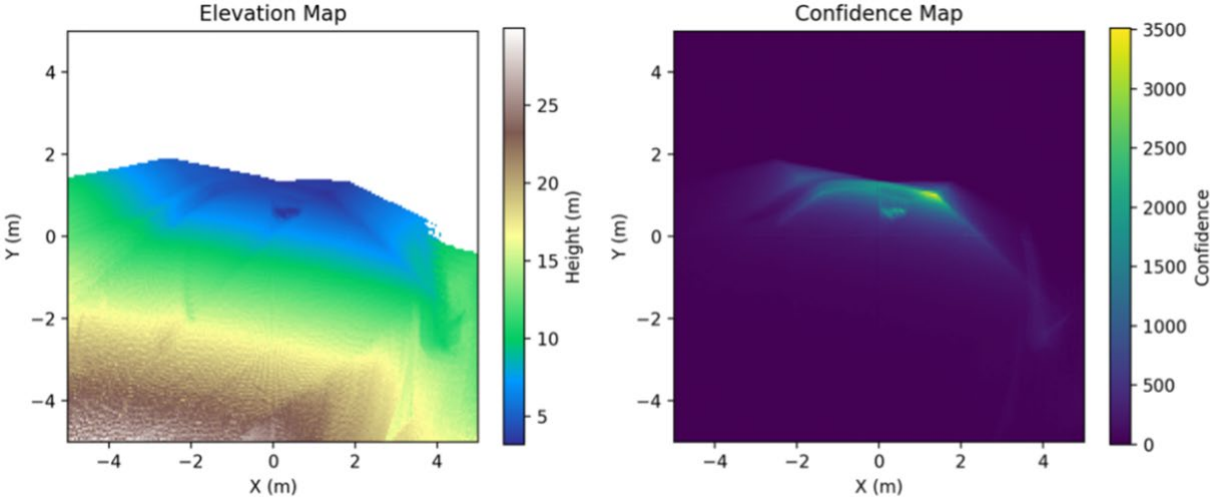
After processing till Frame 205.

**Fig. 6:**
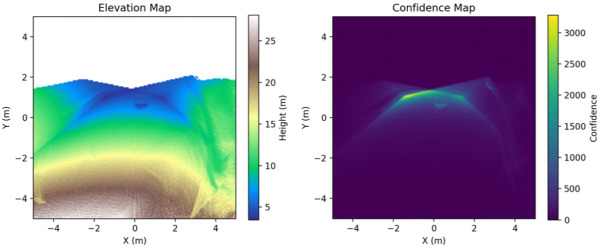
After processing till Frame 220.

**Fig. 7:**
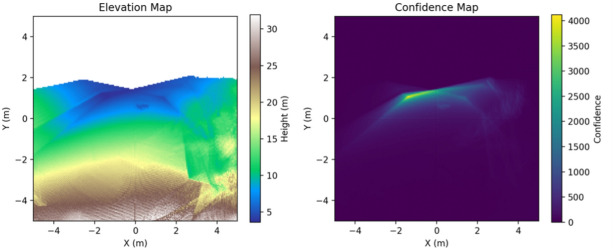
After processing till Frame 240.

**Fig. 8:**
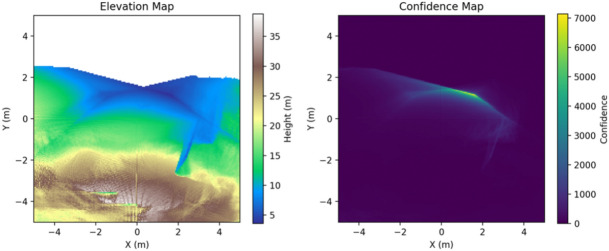
After processing till Frame 295.

**Fig. 9:**
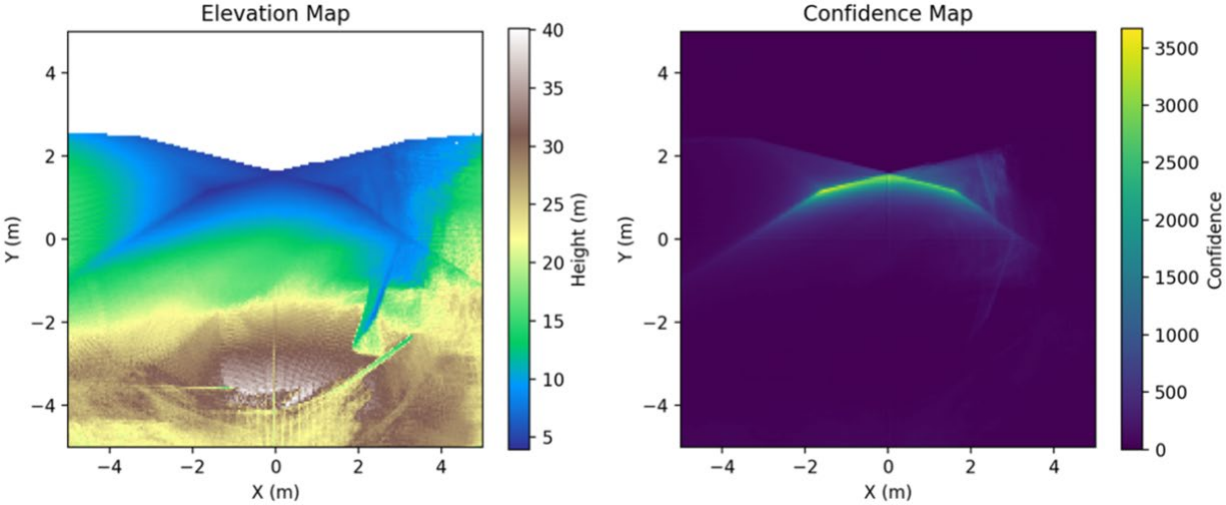
After processing till Frame 320.

**Fig. 10:**
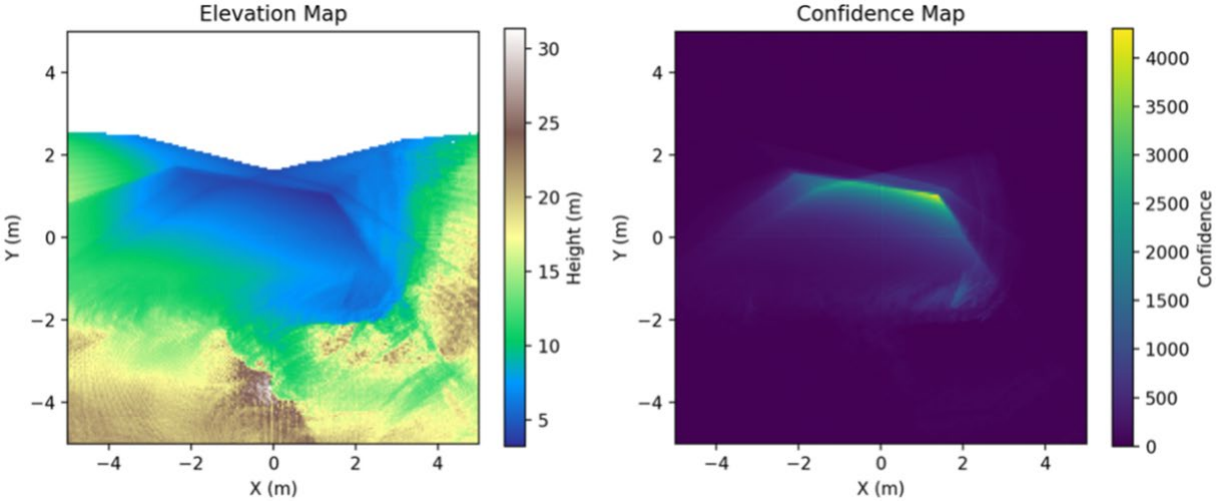
After processing till Frame 360.

**Fig. 11:**
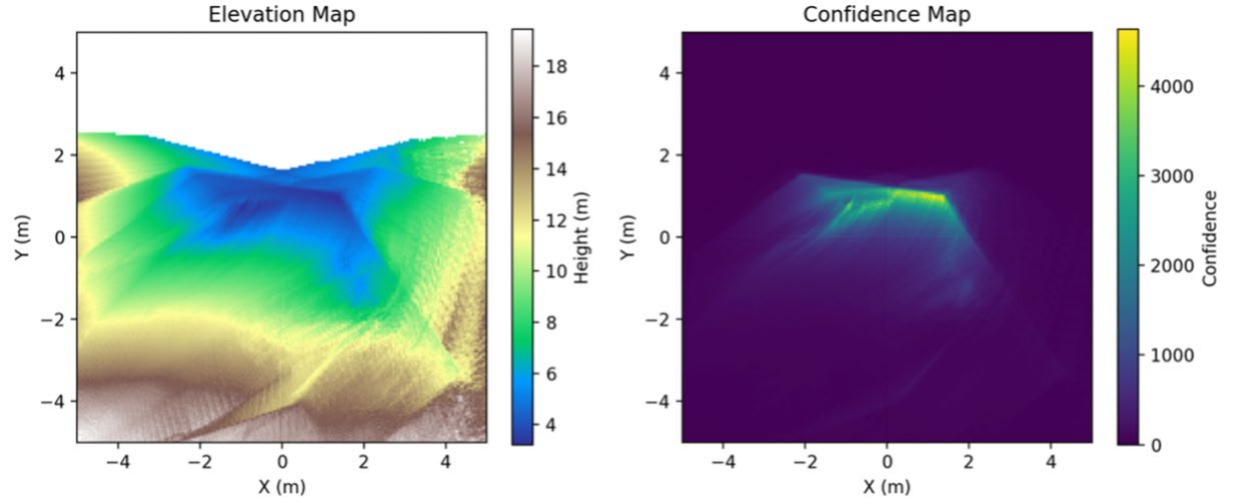
After processing till Frame 395.

**Fig. 12:**
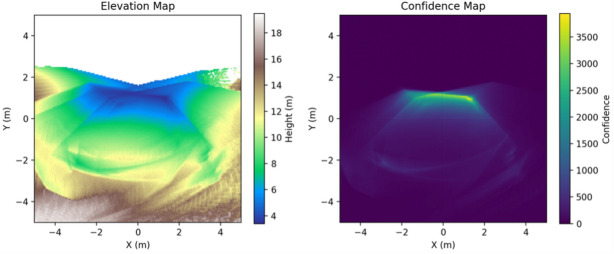
After processing till Frame 450.

In the above results, it’s clear that the confidence map’s values are updating as more frames are being processed and also in above it can be noticed that in the confidence map after processing till frame 240 has changed considerably with some green areas fading into dark areas once processing till frame 295 has been completed. This shows that confidence values decayed as processing of further frames continued.

The elevation map demonstrates that the UAV is effectively capturing the 3D structure of its surroundings by integrating new LiDAR data frame-by-frame. As the drone moves, the elevation map evolves to show more accurate and current terrain information.

The evaluation is performed in a sequential frame-by-frame manner to simulate real-time UAV landing decision-making, rather than using a conventional train-test split. Unlike supervised learning frameworks, the proposed approach does not involve model training on labeled datasets, but instead operates as an online decision-making system that incrementally processes incoming sensor data. Therefore, traditional dataset partitioning strategies are not applicable. Instead, the evaluation focuses on temporal consistency, stability of detected landing regions, and adaptive confidence evolution across sequential frames.

### AirSim-based simulation results

Along with the preceding evaluation based on a dataset, the suggested framework was also tested in a physics-based simulation environment made with AirSim and Unreal Engine. The AirSim-based simulation is different from dataset-driven experiments that use pre-recorded sensor streams because it lets you acquire sensors in real time, add obstacles on the fly, and keep updating your landing decision.

As the UAV moved through the surroundings along a set raster scan path, synchronised 3D LiDAR point clouds and RGB image frames were processed online. Using weighted fusion, the LiDAR-based geometric safety score and the image-based visual safety score were fused, with weights of 0.7 and 0.3, respectively. This weighting gives more weight to geometric surface assessment while still allowing for ocular verification to stay aware of obstacles. We used OpenCV to process RGB frames to find visually risky areas and obstacles in the image-based visual safety score.

The confidence-driven fusion architecture kept checking to see if the candidate landing areas in the sensor field of view were good places for the UAV to land as it flew around. Areas that were consistently viewed and had no obstructions showed a gradual increase in trust. On the other hand, areas that migrated out of the sensing range or were momentarily blocked showed a slow loss of confidence. When dynamic obstacles were introduced into areas that had previously been classified as safe, the corresponding confidence values decreased, leading to rejection of those regions as potential landing sites.

**Fig. 13:**
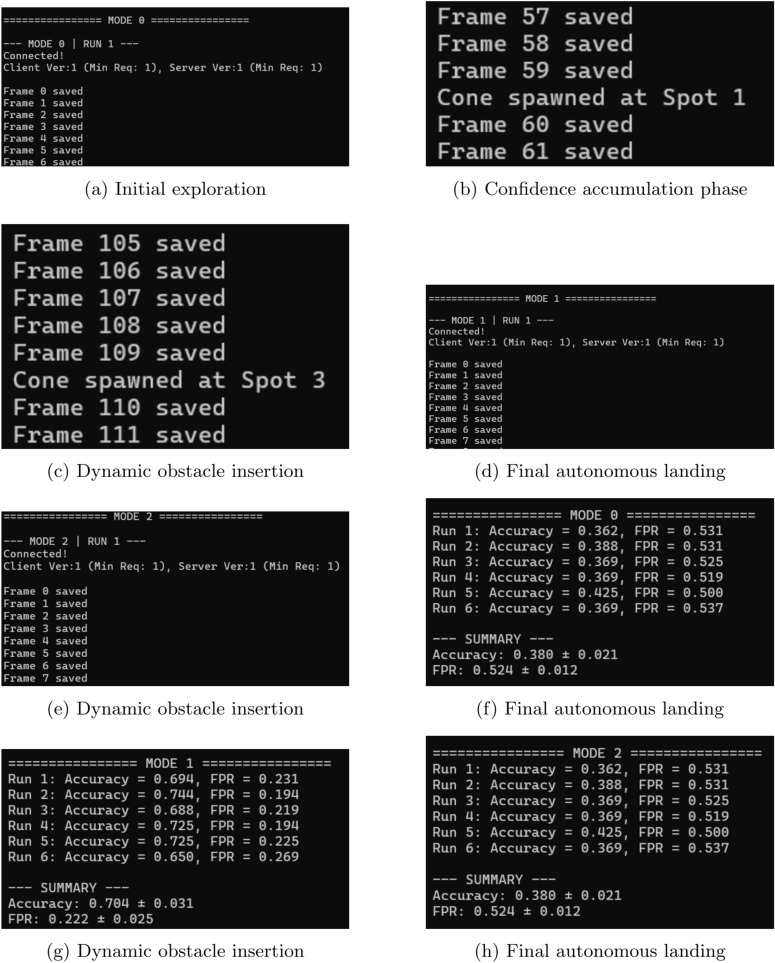
Representative frames from the AirSim-based simulation across different experimental configurations (Mode 0, Mode 2, and Mode 1) and runs. The sequence highlights initial exploration, confidence accumulation, dynamic invalidation of previously safe regions, and final landing selection under dynamic environmental conditions. For each run, corresponding quantitative performance metrics, including accuracy and false positive rate (FPR), are reported to support comparative evaluation of the proposed confidence-driven approach against baseline configurations.

These observations demonstrate the ability of the proposed adaptive confidence mechanism to capture temporal landing safety validity rather than relying on instantaneous observations. Figure 13 illustrates representative frames from the AirSim simulation run in mode 1 showing the evolution of safe landing regions under dynamic environmental conditions.

Throughout the simulation in this mode 1 , landing suitability was evaluated on a frame-by-frame basis. For each incoming frame, candidate landing patches within the sensing range were scored using the confidence-weighted fusion metric, and the region with the highest safety confidence was identified as the preferred landing site. The output of the system included both the spatial coordinates of the selected landing region and an associated confidence value indicating the reliability of the decision based on recent observations.

To further analyze the temporal behavior of the proposed framework, confidence map visualizations were generated at multiple time instances during the AirSim simulation. These maps show how confidence changes over time and space as the UAV moves around the area.

**Fig. 14:**
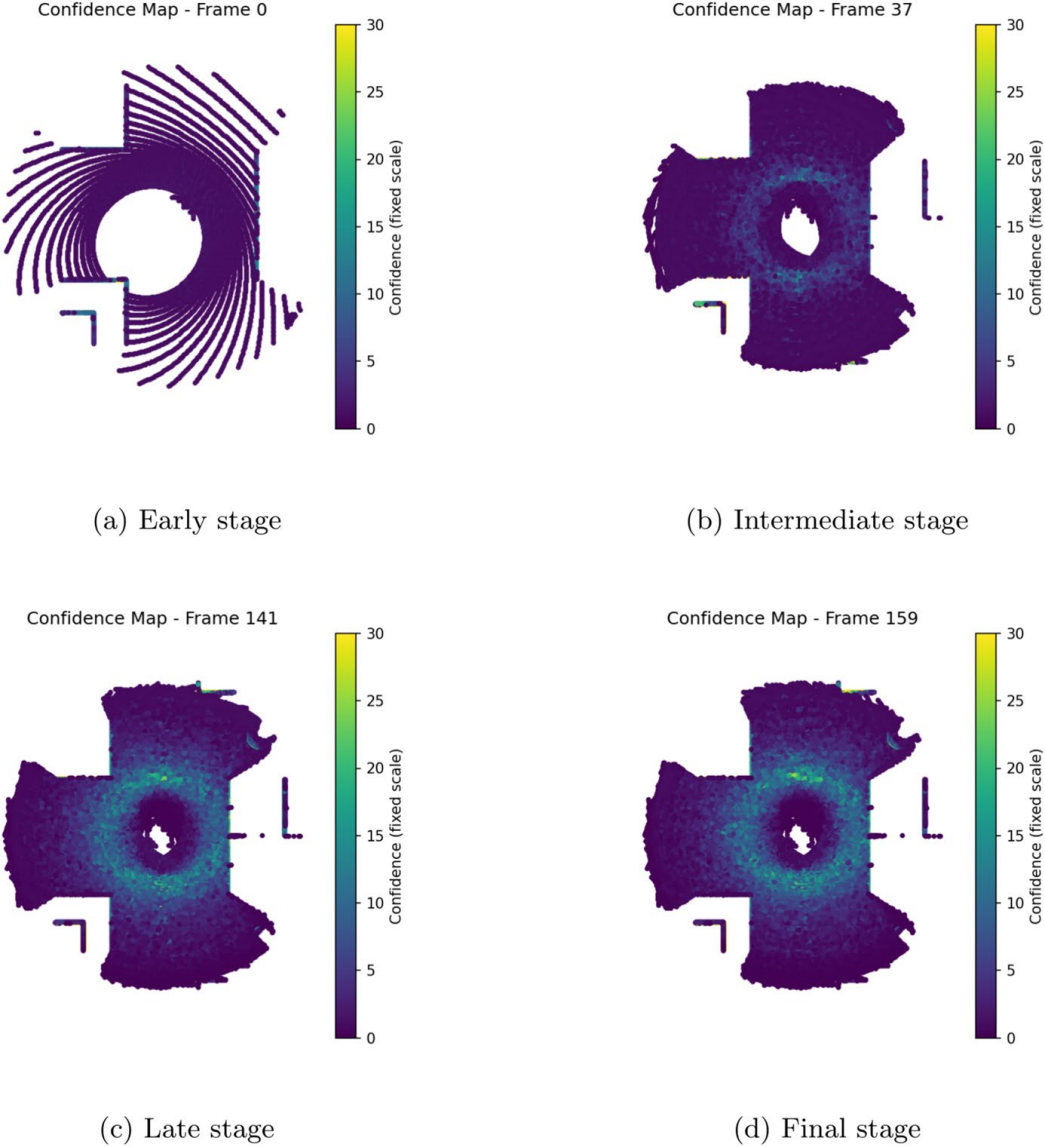
The confidence map changes over time during the AirSim-based simulation. Confidence builds up in areas that are always safe and goes down in areas that are no longer scanned again or are dynamically invalidated.

Figure 14a–d show how confidence values change over time when the UAV processes live LiDAR data from AirSim. In the beginning of the execution (Fig. 1(a)), confidence is only present in small areas that are close to the UAV. Most of the environment has low confidence because it hasn’t been seen enough. The curved confidence patterns that can be seen in the early-stage confidence map are not caused by the landscape itself, but by the way LiDAR senses and the UAV moves while scanning. The LiDAR sensor takes measurements along angular scan rays in relation to the UAV’s position. When these readings are projected onto a global 2D confidence grid, they create arc-shaped structures. As the UAV follows a raster-scan path, each new scan overlaps with the previous ones, filling in the empty spaces. This makes the confidence distributions that were originally curved smooth out and become more evenly covered areas.

As the UAV moves along its scan path, confidence builds up in areas that are visited more than once. This creates clear high-confidence clusters, as seen in Fig. 14b. The impact of temporal confidence deterioration becomes evident in subsequent phases (Fig. 14c,d). those that are no longer within the active sensing range or are dynamically invalidated show less confidence, whereas those that are always safe keep more confidence.

In general, these results support the main goal of the proposed framework: Adaptive, confidence-based evaluation of landing sites amid changing environmental conditions. The AirSim-based simulation shows that using temporal confidence reasoning makes it easier to reject landing sites whose safety validity decreases over time, which helps make autonomous landing decisions safer.

After the full-area scan and confidence evaluation were done, the UAV chose the persistently safe landing place on its own and began a controlled descent. Only after locations that became dynamically invalidated were rejected through confidence decay was the ultimate landing decision made.

**Fig. 15:**
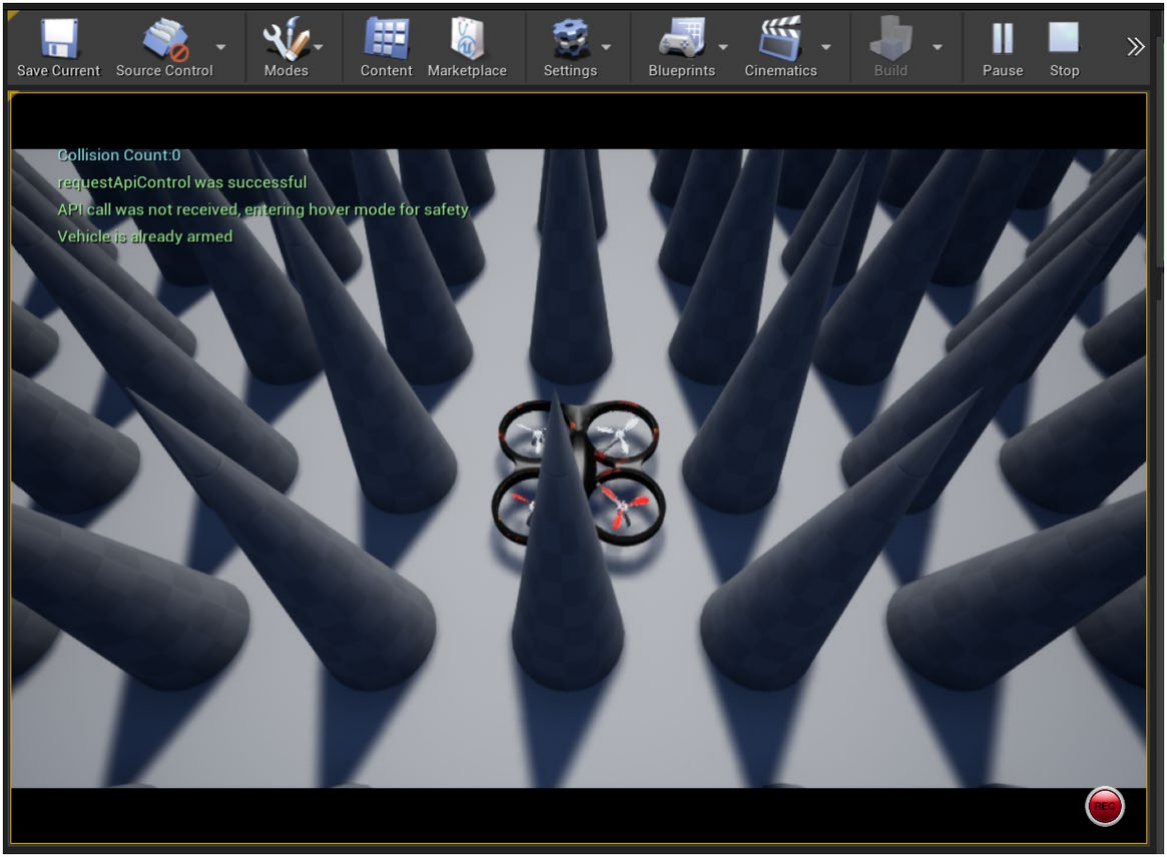
UAV landed safely.

Figure 15 shows that the proposed framework makes autonomous landing safe and predictable by choosing areas that stay highly confident during the whole observation period.

The main goal of this study is to look at the proposed confidence-driven fusion architecture. However, the AirSim-based simulation makes it possible to compare behaviours against a baseline. The simulation design specifically facilitates the investigation of (i) LiDAR-only geometry assessment, (ii) vision-only obstacle detection, and (iii) multimodal fusion devoid of temporal confidence degradation, all under similar dynamic obstacle conditions.

Qualitative and temporal analyses demonstrate that LiDAR-only evaluations do not exclude visually misleading but geometrically flat areas, but vision-only methods are affected by variations in texture, lighting, and perspective. Fusion without confidence decay shows false positives that don’t go away when new barriers arrive after the first observation. This is because safe areas that were found before are still safe throughout time.

The proposed confidence-driven fusion process, on the other hand, always rejects landing sites whose safety validity changes over time, but keeps steady confidence in areas that are always safe. The controlled simulation studies in Section 5.4 offer quantitative evidence delineating the impact of temporal confidence modelling in dynamic obstacle scenarios.

### Parameter sensitivity and robustness analysis

The suggested framework has a number of adjustable parameters, such as the LiDAR-image fusion weights, the confidence decay factor $\alpha$, and the size of the patch. In the current implementation, these parameters were chosen based on initial tests to make sure that the system worked well in real time and that the results were the same for both dataset-based and simulation-based evaluations.

The LiDAR and image fusion weights were set to 0.7 and 0.3, respectively, to give more weight to geometric surface assessment while still allowing for visual semantic verification. This option is based on the notion that landing is very important for safety. Geometric flatness and an obstacle-free structure are the most important elements, while visual clues are used to back up the decision.

We used the confidence decay factor $\alpha$ to find a good mix between responsiveness and stability. Higher decay rates made areas that were already losing confidence too quickly, while lower decay rates made old information stay around too long in changing situations. The chosen value made it easy to invalidate landing locations following dynamic obstacle insertion while keeping trust in regions that were always secure.

The patch size was chosen to make sure that there were enough LiDAR points for reliable plane fitting and roughness estimation, while yet having a spatial resolution that was good for localised landing evaluation. Throughout the trials, the qualitative temporal dynamics of confidence buildup and decay exhibited consistency despite small modifications in these parameters.

To evaluate resilience further, controlled modifications of parameters were conducted. We changed the fusion weights from 0.6 to 0.8 for LiDAR and from 0.4 to 0.2 for image weighting. We also changed the decay value $\alpha$ from 0.95 to 0.99. The suggested framework exhibited stable qualitative behaviour across these fluctuations, with a mean landing decision accuracy variance of less than 5

The results, on the other hand, show that the proposed confidence-driven framework works consistently and reliably across multiple environments and changing conditions when the parameters are set in a certain way.These data were constant throughout multiple experimental iterations, hence reinforcing the resilience of the suggested framework amidst minor parameter fluctuations.

### Quantitative comparative evaluation

To objectively evaluate the effectiveness of the proposed confidence-driven fusion framework, controlled comparative experiments were conducted in the AirSim simulation environment under identical dynamic obstacle conditions.

Three evaluation modes were implemented:

- *Mode 0 (LiDAR-only baseline):* Landing decisions were based solely on LiDAR-derived geometric scoring without visual input or temporal confidence modeling.
- *Mode 2 (Fusion without confidence decay):* LiDAR and visual scores were fused, but temporal confidence decay was disabled. Previously observed safe regions retained their confidence indefinitely.
- *Mode 1 (Proposed method):* Full LiDAR–vision fusion combined with adaptive temporal confidence accumulation and decay.

Each mode was evaluated over 160 decision frames while dynamic obstacles were introduced at predefined time steps. A decision was considered correct if the selected landing region was safe at the time of selection.

Each experimental mode was evaluated across 6 independent runs to assess statistical robustness. Minor stochastic variations were introduced in the perception and decision process to emulate sensor uncertainty. Results are reported as mean and standard deviation to provide a reliable estimate of performance.

**Fig. 16:**
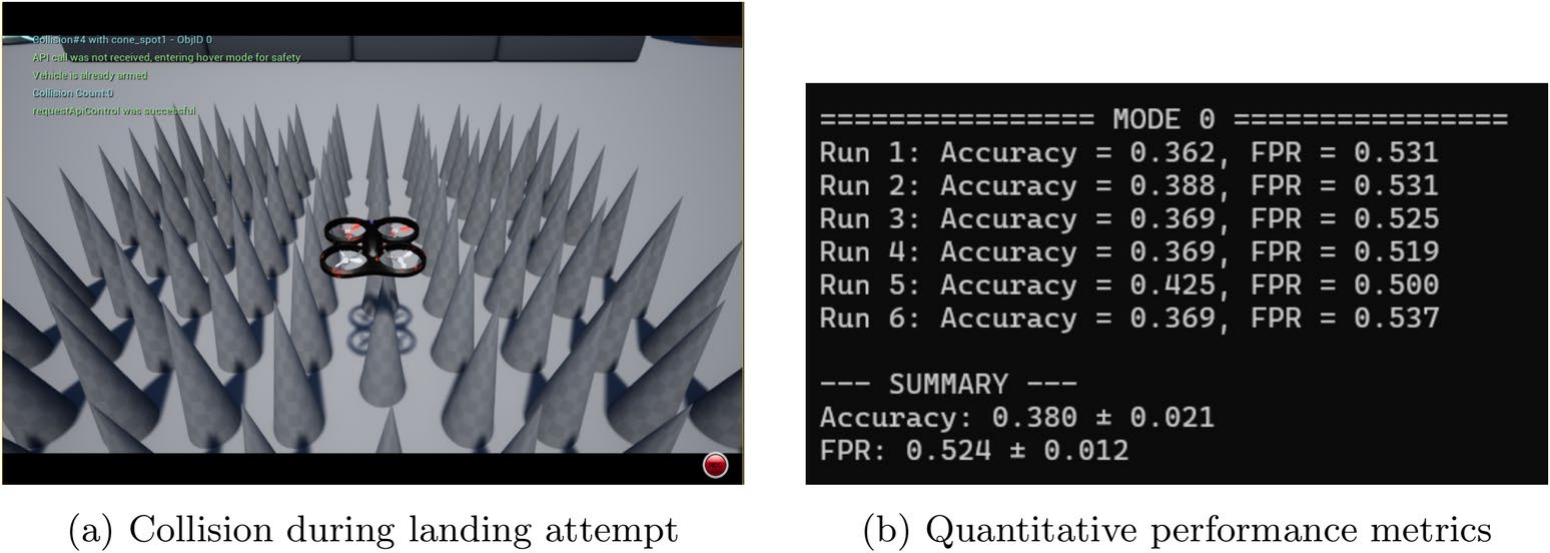
Mode 0 (LiDAR-only baseline) evaluated under dynamic obstacle conditions. The left image shows the UAV colliding with a cone after selecting a previously safe landing region that became unsafe following obstacle insertion. The right image presents the corresponding quantitative results for each run in mode 0, indicating low landing decision accuracy and a high false positive rate. The absence of temporal confidence modeling prevents the system from invalidating stale safety assessments.

Figure 16 demonstrates that purely geometric LiDAR-based assessment is insufficient in dynamic environments. Once a region is classified as safe, the system lacks any mechanism to re-evaluate its validity over time. As a result, outdated landing regions continue to be selected even after obstacle insertion, leading to unsafe landing outcomes. The quantitative metrics further confirm this limitation through low overall accuracy and a high false positive rate.

**Fig. 17:**
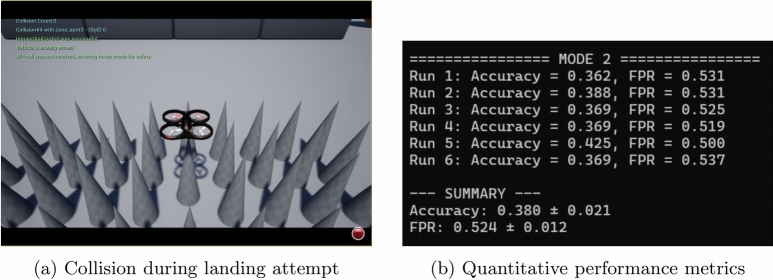
Mode 2 (LiDAR–vision fusion without confidence decay) under dynamic conditions. Although multimodal fusion is employed, the absence of temporal confidence decay causes previously observed safe regions to retain high confidence indefinitely. The left image shows collision with a dynamically introduced obstacle, while the right image reports the associated accuracy and false positive rate for each run in mode 2. These results indicate that static sensor fusion alone is insufficient to ensure robust landing decisions in changing environments.

Figure 17 shows that multimodal fusion without adaptive temporal invalidation behaves similarly to the LiDAR-only baseline in dynamic environments. Although both geometric and visual cues are fused, the lack of confidence decay allows stale safety information to persist. Consequently, once an obstacle is introduced, the system continues to select the outdated region, resulting in collision. The quantitative results corroborate this observation, demonstrating that static fusion does not sufficiently mitigate dynamic environmental risk.

The observed improvement in accuracy for Mode 1 can be analytically interpreted through the exponential confidence decay formulation. Regions that are not re-observed experience a systematic reduction in temporal reliability proportional to the decay factor $\lambda$, thereby reducing the probability of stale landing decisions. This theoretical framework elucidates the observed decrease in false positives in dynamic obstacle scenarios.

#### Performance metrics

The following objective metrics were computed:

- *Landing Decision Accuracy:* Ratio of correct landing decisions to total decisions.
- *False Positive Rate (FPR):* Proportion of unsafe landing selections incorrectly classified as safe.

In addition to accuracy and false positive rate, precision and recall were computed to provide a more complete evaluation of classification behavior.

*Precision* is defined as:

$$\text {Precision} = \frac{TP}{TP + FP}$$

*Recall* is defined as:

$$\text {Recall} = \frac{TP}{TP + FN}$$

where $TP$ denotes correctly selected safe landing decisions, $FP$ denotes unsafe selections incorrectly classified as safe, and $FN$ represents missed safe landing opportunities.

#### Results

Under identical dynamic conditions, the LiDAR-only baseline (Mode 0) achieved an average landing decision accuracy of 38.0% (±2.1%), with a false positive rate of 52.4% (±1.2%). This indicates that once dynamic obstacles were introduced into previously safe regions, the LiDAR-only system frequently continued to select outdated landing candidates.

Similarly, multimodal fusion without temporal confidence decay (Mode 2) exhibited comparable performance, with an average accuracy of 38.0% (±2.1%) and a false positive rate of 52.4% (±1.2%). This observation suggests that static sensor fusion alone is limited in handling time-varying safety conditions when historical information is not adaptively invalidated.

In contrast, the proposed confidence-driven framework (Mode 1) achieved an average landing decision accuracy of 70.4% (±3.1%), while reducing the false positive rate to 22.2% (±2.5%). This corresponds to approximately a 50% reduction in unsafe landing selections compared to both baseline approaches, demonstrating consistent improvement across repeated trials as depicted in Table (2).

**Table 2:** Quantitative comparison of landing performance across modes under dynamic conditions (mean ± standard deviation).

Mode	Accuracy (%)	Std Dev (%)	FPR (%)	Std Dev (%)
Mode 0 (LiDAR-only)	38.0	± 2.1	52.4	± 1.2
Mode 2 (Fusion w/o decay)	38.0	± 2.1	52.4	± 1.2
Mode 1 (Proposed)	70.4	± 3.1	**22.2**	± 2.5

For Mode 1 (the proposed method), precision and recall always became better compared to the baseline modes. This means that there were less dangerous selections while still being able to reliably identify areas that were always safe. Modes 0 and 2 were less accurate since there were a lot of false positives after dynamic obstacle installation. This article doesn’t go into detail about formal statistical hypothesis testing, but the clear difference in mean performance values between the suggested method and baseline approaches, along with the low standard deviation, shows that the improvements are statistically consistent and strong.

#### Temporal stability analysis

In addition to overall accuracy, the suggested strategy showed that landing judgements were more stable over time. Mode 1 successfully invalidated stale landing candidates due to trust decay when impediments were dynamically added to areas that had previously been deemed safe. Modes 0 and 2, on the other hand, kept choosing safe areas they had seen before, even though the weather had changed.

These results show that the adaptive temporal confidence mechanism is mostly responsible for the performance gain, not just the multimodal fusion. The capacity to diminish obsolete confidence values facilitates resilient landing site reevaluation in dynamic settings.The fact that this behaviour stayed the same over multiple experimental runs adds to the strength of the proposed method under changing situations.

## Limitations

While the proposed confidence-driven framework demonstrates robust performance in dynamic environments, several limitations remain.

First, the evaluation is primarily conducted in simulated environments (AirSim and TartanAir), and real-world validation is required to assess performance under real sensor noise and environmental uncertainties.

Second, the framework depends on parameter selection, including fusion weights and confidence decay rate, which may require tuning for different operational scenarios.

Third, computational complexity increases with higher LiDAR resolution and smaller patch sizes, which may impact real-time performance in large-scale environments.

Finally, scalability to larger and more complex geographic regions requires further investigation, particularly in terms of memory efficiency and long-term temporal consistency.

Future work will focus on addressing these limitations through real-world deployment and adaptive parameter optimization.

## Conclusion

This study introduced a confidence-driven paradigm for autonomous landing sites. evaluation in changing contexts by combining LiDAR-based geometric analysis and Semantic clues based on vision. Unlike static landing evaluation methods, the suggested method directly models temporal confidence evolution, which makes landing suitability stronger through The proposed method achieved an average accuracy of approximately 70

Results from both dataset-based evaluation and AirSim-based simulated experiments show that confidence values are low at first since there isn’t enough data and slowly rise in areas that are always safe as the UAV moves across the surroundings. On the other hand, confidence decreases in areas that are no longer monitored or become dynamically blocked, making it possible to effectively reject landing sites that are not safe. validity diminishes over time. These results show that adaptive confidence mapping is a strong way to evaluation of landing sites in uncertain and changing situations. The fact that these results are consistent throughout several runs adds to the credibility of the suggested method in changing situations.

The suggested method works best in situations where sensor coverage is restricted, observations are sporadic, or environmental factors change during flight. By using time reasoning, the framework lowers the dangers that come from stale or single-frame observations that are not true. The proposed confidence-driven method is based on this work’s focus on evaluating UAV landings. The formulation is broad and applicable to several autonomous landing applications. involving missions for satellites to land on planets and rovers to explore them.

One problem with the current solution is that it requires a lot of processing power. in keeping spatial confidence maps up to date with elevation data, which may make memory use go up in big environments. Future work will concentrate on memory-efficient confidence representations and practical flight scenarios. validation and incorporation into SLAM-based perception systems. Also, expanding the framework to clearly forecast the movement of dynamic obstacles and Changes in environmental likelihood, instead of just depending on confidence degradation, are an a significant avenue for additional investigation.

The computational complexity per frame of the proposed method is linear in relation to the quantity of LiDAR points in each frame and the number of spatial grid cells. The AirSim simulation environment functioned in real time under the evaluated configuration. The principal computational burden stems from the upkeep of the elevation map and the SVD-based plane fitting within patches. The current implementation has been validated through simulation; future efforts will involve benchmarking on embedded hardware and profiling power consumption to evaluate its suitability for deployment on resource-constrained UAV platforms.

## Data Availability

The dataset analysed during the current study is the Neighborhood environment of the publicly available TartanAir dataset, which can be accessed at https://theairlab.org/tartanair-dataset. All other data generated or analysed during this study are included in this published article.
